# Analysis of Genomic Alterations Associated with Recurrence in Early Stage HER2-Positive Breast Cancer

**DOI:** 10.3390/cancers14153650

**Published:** 2022-07-27

**Authors:** Yong-Seok Kim, Der Sheng Sun, Juneyoung Ahn, Yongseon Kim, Jung-Sook Yoon, Hye Sung Won

**Affiliations:** 1 Department of Surgery, College of Medicine, The Catholic University of Korea, Seoul 06591, Korea; dydtjr97@catholic.ac.kr (Y.-S.K.); 21000619@cmcnu.or.kr (J.A.); 21600356@cmcnu.or.kr (Y.K.); 2 Department of Internal Medicine, College of Medicine, The Catholic University of Korea, Seoul 06591, Korea; 10301871@cmcnu.or.kr; 3 Clinical Research Laboratory, Uijeongbu St. Mary’s Hospital, College of Medicine, The Catholic University of Korea, Seoul 06591, Korea; yoonjs@catholic.ac.kr

**Keywords:** breast cancer, human epidermal growth factor receptor 2, recurrence, tumor inflammation signature

## Abstract

**Simple Summary:**

This study provides information about the genetic alterations associated with disease recurrence in early stage HER2-positive breast cancer. We found seven upregulated and two downregulated differentially expressed genes in patients with recurrence. In addition, the tumor inflammation signature score and the scores of six immune-related breast cancer signatures were decreased in patients with recurrence. Our study offers insight into the underlying genetic background of recurrence risk and can help to develop effective risk-adapted therapeutic strategies in early stage HER2-positive breast cancer.

**Abstract:**

We aimed to compare gene expression in primary tumors of patients with recurrence and nonrecurrence to gain insight into the biology of high-risk HER2-positive early breast cancer. Patients who underwent curative resection and received adjuvant trastuzumab for HER2-positive early breast cancer were evaluated. Gene expression analyses were performed using NanoString Technologies’ nCounter Breast Cancer 360 Panel. PAM50 intrinsic subtypes and Breast Cancer Signatures including tumor inflammation signature (TIS) were evaluated. Of 247 patients, 28 (11.3%) had recurrence at a median follow-up of 54.2 months. Patients with pathological stage III, tumor size > 5 cm, axillary lymph node metastases, and hormone receptor-negativity were more frequently observed in the recurrent group compared with the nonrecurrent group. In patients with recurrence, seven genes were upregulated significantly, including *WNT11, HAPLN1, FGF10, BBOX1, CXADR, NDP*, and *EREG*, and two genes were downregulated, including *CXCL9* and *GNLY*. TIS score was significantly lower in patients with recurrence compared with controls without recurrence. These findings suggest that activation of oncogenic signaling pathways related to cell proliferation, adhesion, cancer stemness, and noninflamed tumor microenvironment are associated with the risk of recurrence in early stage, HER2-positive breast cancer.

## 1. Introduction

Overexpression of human epidermal growth factor receptor 2 (HER2) protein or amplification of the *HER2 gene*, which is classified as HER2-positive breast cancer, occurs in approximately 15% to 25% of all breast cancers and is associated with a poor prognosis and an increased risk of relapse [1]. The first anti-HER2 targeted agent, trastuzumab, has demonstrated improved outcomes in patients with early stage HER2-positive breast cancer as well as in metastatic disease [1,2]. A meta-analysis of seven randomized trials showed that adding one year of trastuzumab to adjuvant chemotherapy in patients with early stage HER2-positive breast cancer can reduce breast cancer recurrence and mortality by a third [3]. The addition of trastuzumab in the neoadjuvant setting has shown higher rates of pathologic complete response in HER2-positive early breast cancer [4]. Based on these results, the use of trastuzumab as adjuvant or neoadjuvant therapy has become the standard of care for early stage HER2-positive breast cancer. Despite these treatments, however, up to 20% of patients with early stage HER2-positive breast cancer experience recurrence and there is still an unmet need to improve the prognosis for high-risk patients.

With the development of novel anti-HER2 targeted agents, risk-adapted strategies that tailor the intensity of adjuvant or neoadjuvant therapy according to individual risk factors is an emerging area of investigation [5]. Together with this approach, the importance of research on risk factors and resistance mechanisms to identify high-risk patients is growing. Recent advances in molecular biology and genomic analysis have led to a broad understanding of the biology of breast cancer. Many studies have demonstrated that various factors, including intrinsic molecular subtypes, genomic alterations, and tumor microenvironments influence aggressive tumor behavior and response to therapy [6,7,8,9]. An approach that integrates genomics into clinicopathological risk factors enables better prediction of outcomes in patients at high risk for breast cancer. Multiple gene expression signatures are already being used in breast cancer risk classification and decision-making for early stage hormone receptor (HR)-positive breast cancer [10,11,12]. From this standpoint, the primary objective of our study was to identify genomic alterations associated with disease recurrence in HER2-positive early breast cancer. We analyzed the clinicopathological characteristics of recurrent tumors and explored the genomic factors affecting recurrence through gene expression analysis in relapsing and nonrelapsing patients with HER2-positive early breast cancer who received adjuvant trastuzumab.

## 2. Materials and Methods

### 2.1. Patients

The patients who underwent curative resection for HER2-positive early breast cancer in our hospital between March 2010 and December 2018 were evaluated. The inclusion criteria were as follows: (1) HER2-positive breast cancer defined as tumors with an immunohistochemistry (IHC) score of 3+ for HER2 staining or an IHC score of 2+ with HER2 gene amplification by in situ hybridization assay; (2) all planned adjuvant treatment with chemotherapy and trastuzumab had been completed, and (3) availability of clinical data with follow-up.

In total, 247 patients were included in this study, comprising 219 patients with nonrecurrence and 28 patients with recurrence. The following clinicopathological data were collected retrospectively: age, sex, body mass index, menopausal status, family history of breast cancer, smoking history, comorbidity, date of breast cancer diagnosis, date of surgery, types of surgery, pathological stage according to the American Joint Committee on Cancer staging system (7th edition), histological types, tumor location, tumor size, number of positive lymph nodes (LNs), histological grade, nuclear grade, lymphovascular invasion, estrogen receptor (ER) status, progesterone receptor (PR) status, HER2 status, and Ki-67 labeling index, adjuvant chemotherapy, adjuvant endocrine therapy, adjuvant radiation therapy, recurrence, date of recurrence, survival, date of death, and cause of death.

### 2.2. Gene Expression Analysis

We analyzed gene expression profiles of 34 primary tumor tissues, 26 (76.5%) from patients with recurrence, and 8 (23.5%) from controls without recurrence who were considered as high risk by clinicopathological prognostic variables (Supplementary Table S1). Total RNA was isolated from formalin-fixed, paraffin-embedded archival tumor samples. Gene expression analyses were performed at PhileKorea Technology (Seoul, Korea), using the nCounter Breast Cancer 360 Panel (NanoString Technologies, Seattle, WA, USA) according to the manufacturer’s instructions. The nCounter Prep Station and nCounter Digital Analyzer (NanoString Technologies) were used to read the samples. The Breast Cancer 360 Panel includes 776 individual genes across 23 key breast cancer pathways and provides molecular intrinsic subtypes based on the PAM50 classifier algorithm [13].

Raw data were quality controlled and normalized using positive control probes and 18 housekeeping genes in nSolver Analysis Software (v. 4.0; NanoString Technologies). All samples passed the quality control test. Changes in gene expression were presented as fold changes compared to the control group and expression values were log2-transformed for statistical analysis. Genes were considered to be differentially expressed if they had an absolute value of log2-fold change ≥2.0 at a false discovery rate of <0.05. Heatmaps of gene expression profiles were created using hierarchical clustering and all data analysis was performed with nSolver Analysis Software.

The data were sent to NanoString Technologies Inc. for additional analysis to identify PAM50 intrinsic subtypes and Breast Cancer 360 Signatures including tumor inflammation signature (TIS) [13]. Data from 29 samples that passed predefined quality control thresholds were included in these analyses. Tumors were classified into one of four intrinsic subtypes, i.e., luminal A, luminal B, basal-like, and HER2-enriched, based on the PAM50 classifier algorithm using the 50-gene subtype predictor as described by Parker et al. [14]. Breast Cancer 360 Signatures comprise about 40 signatures across 13 categories of breast cancer biology with well-established roles in breast cancer and immuno-oncology. Signature scores were calculated using prespecified established algorithms developed by NanoString Technologies. The TIS is a validated 18-gene classifier correlating distinct immune-related signatures associated with clinical benefits of immune checkpoint inhibitors (ICIs) [15]. A TIS score is generated through weighted linear combination of these gene expression values against the expression of stable housekeeping genes, reflecting the presence of a peripherally suppressed adaptive immune response in the tumor microenvironment, including antigen presentation, chemokine expression, cytotoxic activity, and adaptive immune resistance.

### 2.3. Statistical Analysis

Categorical variables were compared using chi-square and Fisher’s exact tests. The significance of differences in continuous variables was evaluated using Student’s *t*-test and Mann–Whitney *U* test. Overall survival (OS) was defined as the time from the date of surgery to the date of death from any cause or the date of last follow-up. Recurrence-free survival (RFS) was defined as the time from the date of surgery to the date of first recurrence or death from any cause. NanoString data were analyzed for statistical significance using nSolver Analysis Software. Clinical data and box plots of signature scores with associated *p*-value calculations were analyzed using SPSS software (v. 18; SPSS, Chicago, IL, USA) and *p* < 0.05 was considered significant.

## 3. Results

### 3.1. Clinicopathological Characteristics and Recurrence Patterns

Median follow-up time was 54.2 months (range, 1.6–134.3 months). Of the 247 patients, 28 (11.3%) patients had recurrences during the follow-up period. The differences in clinicopathological characteristics between patients without recurrence and those with recurrence are summarized in Table 1.

The proportion of HR-negative breast cancer was higher in patients with recurrence than in those without recurrence (*p* = 0.013). Patients with pathological stage III, tumor size > 5 cm, and axillary LNs metastases were more frequently observed in the recurrent group compared with the nonrecurrent groups (*p* = 0.007, 0.023, and 0.001, respectively). The frequency of tumors with a Ki-67 labeling index above 20% tends to be higher in the recurrent groups.

The median interval from the last dose of adjuvant trastuzumab to recurrence was 8.7 months (range, 0.1–88.4 months). No difference was noted in the trastuzumab treatment sequence, i.e., sequential or concurrent incorporation of trastuzumab between patients with recurrence and those without recurrence. The number of sites and involved organs varied among patients. Fifteen patients (53.6%) had a single site of recurrence, and five (17.9%) patients had a recurrence at three or more sites. The site of first recurrence was most commonly the lung (n = 15, 53.6%), followed by the LNs (n = 11, 39.3%), bone (n = 8, 28.6%), chest wall (n = 3, 10.7%), brain (n = 3, 10.7%), breast (n = 2, 7.1%), liver (n = 1, 3.6%), and skin (n = 1, 3.6%). Biopsy at the site of recurrence was performed in 21 of 28 patients with recurrence, and subtype switching was observed in 10 cases (47.6%). Gain of ER expression was observed in two patients, and one patient showed a gain of ER and loss of PR expression. Loss of HER2 expression was observed in one and four patients with HR-negative and HR-positive tumors, respectively. Loss of both ER and HER2 expression was observed in two patients. In other words, seven (33.3%) of twenty-one patients who underwent a biopsy at the site of recurrence showed loss of HER2 expression.

The median RFS of patients without recurrence and those with recurrence was 54.4 and 25.5 months, respectively. The three-year OS rates for patients without recurrence and with recurrence were 100% and 78.5%, respectively.

### 3.2. Differentially Expressed Genes between Recurrence and Nonrecurrence Groups

**Figure 1:**
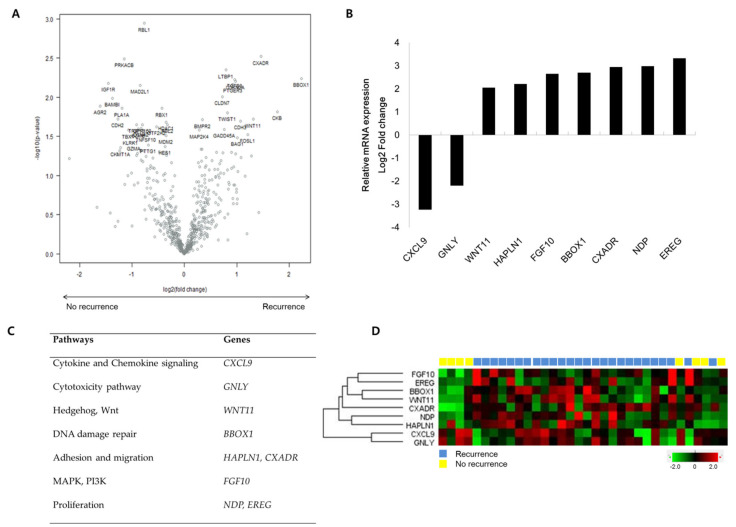
nCounter Breast Cancer 360 Panel analysis (n = 34). (**A**) Volcano plot showing differentially expressed genes comparing nonrecurrence and recurrence groups. (**B**) Relative mRNA expression of nine genes with an absolute value of log2-fold change ≥ 2.0 and *p*-value ≤ 0.05. (**C**) Nine genes significantly differentially expressed in nonrecurrence and recurrence groups to categorize the biological processes. (**D**) Heatmap of NanoString gene expression analysis by nine genes associated with recurrence. The heatmap indicates upregulation (red), downregulation (green), and mean gene expression (black).

When comparing gene expression using Breast Cancer 360 Panel, we identified nine differentially expressed genes (DEGs) in patients with recurrence compared to those without recurrence. Seven genes including *WNT11, HAPLN1, FGF10, BBOX1, CXADR, NDP,* and *EREG* were significantly upregulated, and two genes including *CXCL9* and *GNLY* were downregulated in patients with recurrence (Figure 1A–D).

The upregulated genes were mainly enriched in oncogenic signaling pathways related to cell proliferation, adhesion/migration, and cancer stemness, while downregulated genes were enriched for immune response to the tumor microenvironment.

WNT11 is a positive regulator of the noncanonical Wnt signaling pathway, which plays a role in carcinogenesis including cell proliferation and migratory capacity of cancer cells [16,17]. Norrin cystine knot growth factor (NDP) is a cysteine-rich growth factor required for angiogenesis in various organs. It can activate the canonical Wnt/β-catenin signaling pathway via interaction with Wnt receptors in some cancers [18]. Fibroblast growth factor 10 (FGF10) is a multifunctional mesenchymal–epithelial signaling growth factor, which activates several intracellular signaling pathways such as PI3K/Akt or MAPK/ERK pathways, resulting in cell proliferation, differentiation, and invasion [19]. Epiregulin (EREG) is a member of the epidermal growth factor (EGF) family and can function as a ligand of the EGF receptor (EGFR). The EREG/EGFR pathway is known to play a role in cancer pathogenesis by regulating cell differentiation and growth [20]. Gamma-butyrobetaine hydroxylase 1 (BBOX1) is a critical enzyme that facilitates cell synthesis of carnitine, which is important for energy metabolism [21]. Hyaluronan and proteoglycan link protein 1 (HAPLN1) is a component of the extracellular matrix proteins and promotes tumor invasion through the extracellular matrix remodeling [22]. Coxsackie virus and adenovirus receptor (CXADR) is an adhesion molecule known for its role in virus–cell interactions and organogenesis [23].

The chemokine induced by interferon-gamma, known as CXCL9 is one of the ligands that bind to the chemokine receptor, CXCR3. CXCL9 is known as a mediator of immune cell recruitment and plays an important role in the antitumor immune response. Granulysin (GNLY) is a cytolytic and proinflammatory molecule present in cytotoxic granules together with perforin and granzymes. It is expressed by cytotoxic T cells and natural killer cells and acts as an immune effector or cytotoxic factor that can kill a variety of microbes and tumor cells [24].

### 3.3. PAM50 Intrinsic Subtypes and Tumor Inflammation Signature

PAM50 intrinsic subtypes were determined on tumor tissues included in gene expression analysis according to the 50-gene subtype predictor using the NanoString nCounter Analysis System. We confirmed the heterogeneity of intrinsic subtypes in HER2-positive breast cancers. Among 21 patients with recurrence, 11 (52.4%), six (28.6%), two (9.5%), and two (9.5%) were identified as HER2-enriched, luminal A, luminal B, and basal-like, respectively. Eight controls without recurrence showed four HER2-enriched (50%), three luminal B (37.5%), one luminal A (12.5%), and no basal-like subtypes. Interestingly, the discordance between HR status by IHC and PAM50 intrinsic subtype was more frequent in patients with recurrence. Among twenty-one patients with recurrence, ten HR-negative/HER2-positive tumors (71.4%) were identified as HER2-enriched subtype (two luminal [14.3%] and two basal-like [14.3%]), and six HR-positive/HER2-positive tumors (85.7%) were identified as a luminal subtype (one HER2-enriched [14.3%]). On the other hand, all three HR-negative/HER2-positive tumors showed HER2-enriched subtype and four of five HR-positive/HER2-positive tumors (80%) were identified as luminal subtype (one HER2-enriched [20%]) subtype in eight controls without recurrence.

**Figure 2:**
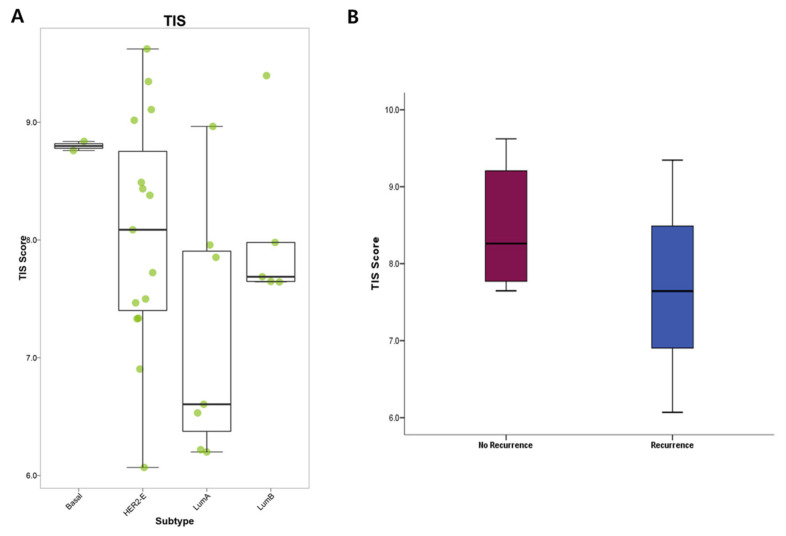
Comparative boxplots of tumor inflammation signature (TIS) score. (**A**) TIS score was higher in the basal-like and HER2-enriched subtypes than in the luminal subtypes. (**B**) TIS score was significantly lower in patients with recurrence (blue) compared to controls without recurrence (red) (*p* = 0.046). Mann–Whitney *U* test.

The TIS is associated with the presence of tumor immunogenicity and high TIS scores indicate an inflamed tumor phenotype. We found that TIS score was higher in the basal and HER2-enriched subtypes than in the luminal subtypes (Figure 2A). Interestingly, the TIS score was significantly lower in patients with recurrence compared with controls without recurrence (mean ± standard deviation: 7.7 ± 0.9 vs. 8.5 ± 0.8, *p* = 0.046) (Figure 2B).

**Figure 3:**
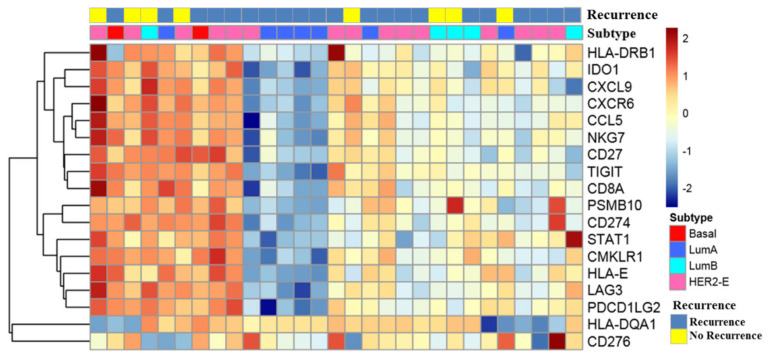
Heatmap for the 18 genes in the tumor inflammation signature (TIS). Heatmap showing the individual TIS genes normalized expression, intrinsic molecular subtypes, and recurrence. The heatmap indicates upregulation (red), downregulation (blue), and mean gene expression (yellow). The columns represent individual tissue samples: luminal A (blue), luminal B (sky blue), basal-like (red), and HER2-enriched (pink). The rows represent individual genes.

The heatmap of 18 individual genes included in the TIS score, intrinsic subtypes, and recurrence are shown in Figure 3. This is consistent with the results of gene expression analysis, i.e., *CXCL9* and *GNLY*, which are indicative of antitumor immune response, were downregulated in patients with recurrence.

**Figure 4:**
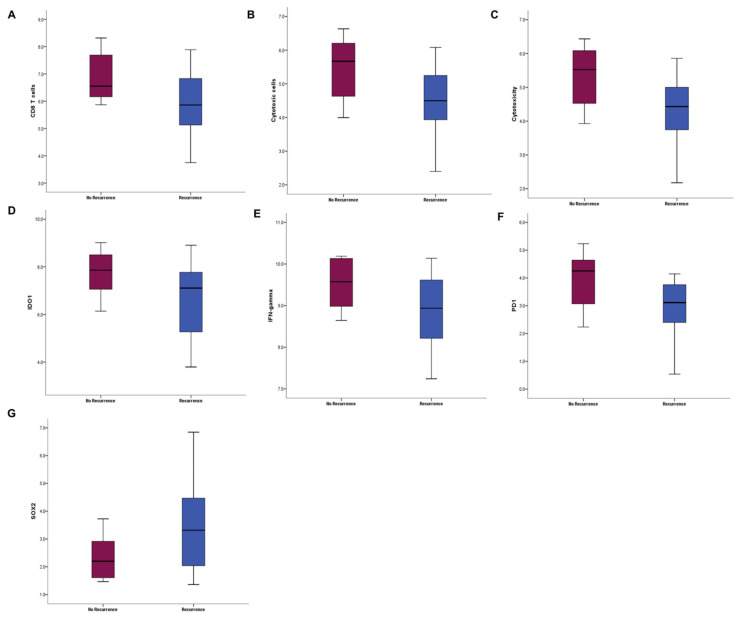
Comparative boxplots of seven breast cancer signature scores, which were statistically different between patients with recurrence (blue) and without recurrence (red). (**A**) CD8 T cells, (**B**) cytotoxic cells, (**C**) cytotoxicity, (**D**) indoleamine 2,3-dioxygenase 1 (IDO1), (**E**) interferon-gamma, (**F**) programmed cell death protein 1 (PD1), and (**G**) SRY-box transcription factor 2 (SOX2). Mann–Whitney *U* test.

Among other breast cancer signatures, seven signatures were found to be statistically different between patients with recurrence and without recurrence: CD8 T cells, cytotoxic cells, cytotoxicity, indoleamine 2,3-dioxygenase 1 (IDO1), interferon-gamma, programmed cell death protein 1 (PD1), and SRY-box transcription factor 2 (SOX2) (Figure 4A–G, Supplementary Table S2). The scores of six signatures related to tumor immune microenvironment were decreased in patients with recurrence compared with controls without recurrence. Meanwhile, the score of SOX2, known as a key driver in cancer stemness, was increased in patients with recurrence compared with controls without recurrence. We observed that six immune-related signatures, except SOX2, showed a significant positive correlation with the TIS score (Supplementary Figure S1A–F).

## 4. Discussion

Recent evidence suggests that clinical prognostic parameters combined with a genomic assay can improve the prediction of recurrence risk across all breast cancer subtypes [10,25,26,27]. We identified seven upregulated and two downregulated DEGs in patients with recurrence in addition to traditional clinicopathological prognostic factors, including pathological stage, tumor size, nodal status, and HR status. The upregulated genes, including *WNT11, HAPLN1, FGF10, BBOX1, CXADR, NDP,* and *EREG* are associated with tumor proliferation, migration, and cancer stemness. The downregulated genes, including *CXCL9* and *GNLY,* are associated with antitumor immunity. These genomic findings are consistent with the current understanding of breast cancer biology and with earlier studies demonstrating an adverse outcome following activation of oncogenic signaling pathways, and a favorable outcome with immune infiltration and activation of cytotoxic T cells [27,28]. Abnormal activation of the Wnt signaling pathway is known to play a crucial role in the pathogenesis of several cancers including breast cancer [16]. Previous studies have shown that upregulation of Wnt11 increases the migratory capacity of breast cancer cells and upregulating the Wnt signaling pathway is associated with trastuzumab-resistant breast cancers [17,29]. The EGFR pathway activated by EREG and the PI3K/Akt/mTOR pathway activated by FGF10 are well known to be part of the mechanisms of resistance to HER2-targeted therapy in HER2-positive breast cancer [28,30,31]. Cell adhesion molecules such as HAPLN1 and CXADR have been reported to contribute to tumorigenesis in several ways [22,32,33]. CXADR is known as a key regulator of adhesion and inflammation and has been shown to have both oncogenic and tumor suppressive functions depending on the type of cancer. Although precise mechanistic pathways in cancer remains poorly understood, the emerging role of CXADR in tumorigenesis has been reported, including control of cell–cell adhesion, recruitment of immune cells, and inducer of the epithelial to mesenchymal transition. CXADR expression has also been associated with cancer stem cell phenotype and resistance to chemotherapy [22,32,33]. Liao et al. reported that BBOX1 was essential for sustaining mitochondrial function and activating mTORC1-mediated glycolysis required for cancer cell proliferation and tumorigenesis in triple-negative breast cancer [21]. These reports support the notion that the genetic alterations upregulated in our study are associated with worse prognosis and further validation is required to confirm that these gene signatures are potential biomarkers for recurrence in early stage HER2-positive breast cancer.

We also identified a prognostic role for immune activation characterized by immune-related genes such as chemokine (*CXCL9*) and cytotoxic granules (*GNLY*) in HER2-positive early stage breast cancer. Interestingly, this finding was in line with TIS score and breast cancer signatures. That is, patients with recurrence showed a lower score of both TIS and six immune signatures, indicating a noninflamed tumor. Although the role of ICIs in HER2-positive breast cancer remains unclear, many studies suggest that tumor immunogenicity plays an important role in treatment response and prognosis in HER2-positive breast cancer, as well as triple-negative breast cancer [34,35]. Tumor-infiltrating lymphocytes (TILs), tumor mutation burden, and expression of immune checkpoints, such as programmed death-ligand 1 (PD-L1), represent surrogate biomarkers for the tumor immunogenicity [36]. A number of studies have shown that higher levels of TILs are associated with achieving a pathological complete response (pCR) as well as improving survival in patients with HER2-positive breast cancer [36,37,38,39]. Additionally, higher PD-L1 expression in breast cancer is positively associated with high TILs, and patients with higher PD-L1 expression show higher pCR rate and better survival outcomes [36]. Previous studies described how immune gene signatures can be considered as another predictor of benefit from HER2-targeted agents. Fernandez-Martinez et al. reported that gene expression signatures related to intrinsic subtype and immune activation contribute to predicting pCR and survival outcomes using tumor gene expression profiles from Cancer and Leukemia Group B 40601, a dual HER2 blockade added to neoadjuvant chemotherapy in HER2-positive breast cancer [8]. Another study from the American College of Surgeons Oncology Group Z1041 trial showed that activated immune signatures, including T-cell, B-cell, and inflammatory signatures were enriched in cases that achieved a pCR in HER2-positive breast cancer patients who received neoadjuvant chemotherapy and trastuzumab [40]. Recently, the HER2DX risk score was presented as the first assay to integrate clinical data with genomic data to predict prognosis in early stage HER2-positive breast cancer, which is in line with our data. Prat et al. reported that the HER2DX combined prognostic score, consisting of tumor size, nodal status, TILs, intrinsic molecular subtype, and 13 individual genes, was significantly associated with disease-free survival in early stage HER2-positive breast cancer [26]. Subsequently, Prat et al. developed a new HER2DX risk score by including immune-related genes. The new risk score was based on two clinical data points, including tumor size and nodal stage, and four different gene signatures, namely immune infiltration, tumor cell proliferation, luminal differentiation, and HER2 amplicon expression [41]. Among the HER2DX variables, immune infiltration and luminal differentiation were associated with good risk outcomes, whereas tumor cell proliferation, tumor size, and nodal stage were associated with poor risk outcomes.

In this study, we focused on the potential of CXCL9 and TIS scores as immune signatures to identify patients at high risk for HER2-positive breast cancer, especially because both of these biomarkers are well known as predictors of ICI responses. A large-scale meta-analysis showed that clonal tumor mutation burden and CXCL9 expression were the strongest predictors of ICI responses across seven tumor types [42]. Liang et al. reported that elevated levels of CXCL9 mRNA were associated with favorable outcomes in HR-negative breast cancer and CXCL9 was correlated with immune cell infiltration and immune-related biomarkers [43]. TIS score was developed as a predictive biomarker of ICIs from a large cohort of pembrolizumab-treated patients across nine different tumor types and the predictive utility of TIS score has been verified in many other studies [15,44,45,46]. Schroth et al. reported that two gene signatures including BRCAness and TIS score, were associated with clinical outcome or presence of tumor immunogenicity in HR-positive early breast cancer [46]. TIS scores were strongly associated with stromal TIL infiltrates, but TIS and TIL features showed only partial overlap [46].

Preclinical studies showed that the combination of ICIs and trastuzumab could improve therapeutic activity in HER2-positive breast cancer [47]. However, the efficacy of ICIs combined with HER2-targeted therapy in HER2-positive advanced breast cancer seems to be unsatisfactory, unlike the significant roles of ICIs in advanced or early stage triple-negative breast cancer [34,36]. Several clinical trials with ICIs are currently ongoing in HER2-positive early breast cancer with the rationale that it is better to anticipate ICIs as early as possible in the course of disease [34]. To obtain more satisfactory outcomes with immunotherapy in HER2-positive breast cancer, reliable biomarkers and smart therapeutic strategies tailored to individual tumor biology and composite biomarkers are the most important factors, and our study can be used as a reference for planning these subsequent studies. This study has several limitations. This was a retrospective study with a relatively short follow-up period. Due to the pilot nature of the study, the number of patients included in the genomic analysis was small; thus, our findings must be interpreted with caution and require validation in larger studies. This study did not reflect racial and ethnic diversity; hence, care must be taken in generalizing these results.

## 5. Conclusions

We found several oncogenic driver alterations and immune-related markers associated with recurrence in early stage HER2-positive breast cancer. These alterations suggest that prognosis can be refined by incorporating clinical factors in conjunction with genomic alterations. Further studies will be required to best integrate clinicopathological factors and molecular genomic variables for optimizing risk stratification. Validation is needed of the role of immune-related markers that can provide predictive information for ICIs in early stage breast cancer. This is expected to enable personalized, risk-adapted therapy with new potential targeted drugs and consequently, improve the prognosis of patients with early stage HER2- positive breast cancer.

## Figures and Tables

**Table 1:** Clinicopathological characteristics according to recurrence.

Variables	Nonrecurrence (n = 219) No. (%)	Recurrence (n = 28) No. (%)	*p* Value
Age, Median (range)	50 (24–77)	48 (35–65)	0.230
Menopausal status			0.732
Premenopause	102 (46.6)	14 (50.0)	
Postmenopause	117 (53.4)	14 (50.0)	
BMI, kg/m^2^			0.285
<25	125 (57.0)	13 (52.0)	
≥25	94 (43.0)	15 (48.0)	
Presence of FHx	9 (4.1)	1 (3.5)	0.892
Smoking			0.003
Non- or Ex-smoker	210 (95.9)	23 (82.1)	
Current smoker	9 (4.1)	5 (17.9)	
Presence of comorbidity	67 (30.6)	12 (42.9)	0.190
Tumor location			0.276
Right	105 (47.9)	16 (57.1)	
Left	112 (51.2)	11 (39.3)	
Both	2 (0.9)	1 (3.6)	
Histology			0.231
Invasive ductal	198 (90.4)	28 (100.0)	
Invasive lobular	4 (1.8)	0	
Others	17 (7.8)	0	
Hormone receptor status			0.013
HR-negative	87 (39.7)	18 (64.3)	
HR-positive	132 (60.3)	10 (35.7)	
Pathological stage			0.007
Stage 0	8 (3.6)	0	
Stage I	69 (31.5)	6 (21.4)	
Stage II	107 (48.9)	10 (35.8)	
Stage III	35 (16.0)	12 (42.8)	
Tumor size			0.023
≤5 cm	210 (95.9)	24 (85.7)	
>5 cm	9 (4.1)	4 (14.3)	
LN status			0.001
LN-negative	133 (60.7)	8 (28.6)	
LN-positive	86 (39.3)	20 (71.4)	
LNR, mean ± SD	0.16 ± 0.64	0.28 ± 0.27	0.093
Histological grade			0.453
G1	27 (12.3)	2 (7.1)	
G2	60 (27.4)	11 (39.3)	
G3	126 (57.6)	15 (53.6)	
Unknown	6 (2.7)	0	
Presence of LVI	109 (49.8)	20 (71.4)	0.095
Ki-67 labeling index *			0.073
<20%	75 (35.9)	5 (18.5)	
≥20%	134 (64.1)	22 (81.5)	
Type of Operation			0.001
BCS	133 (60.7)	5 (17.9)	
TM	86 (39.3)	23 (82.1)	
Type of Adjuvant CTx			0.070
Anthracycline-based	23 (10.5)	3 (10.7)	
Anthracycline/taxane	82 (37.4)	14 (50.0)	
Non-anthracycline	114 (52.1)	11 (39.3)	
Adjuvant Endocrine Tx	132 (60.3)	10 (35.7)	0.013
Adjuvant Radiotherapy	177 (80.8)	20 (71.4)	0.244

BMI: body mass index; FHx: family history; HR: hormone receptor; LN: lymph node; LNR: lymph node ratio; SD: standard deviation; LVI: lymphovascular invasion; BCS: breast conserving surgery; TM: total mastectomy; CTx: chemotherapy; Tx: therapy. * This variable has missing data.

## Data Availability

The data presented in this study are available from the corresponding author on reasonable request.

## Supplementary Materials

The following supporting information can be downloaded at: https://www.mdpi.com/article/10.3390/cancers14153650/s1, Table S1: Clinicopathological characteristics of eight controls without recurrence included in the gene expression analysis. Table S2: Breast Cancer Signature scores between patients with recurrence and without recurrence; Figure S1: Correlation of TIS score and six immune-related Breast Cancer Signature scores, which were statistically different between patients with recurrence and without recurrence. (A) CD8 T cells, (B) cytotoxic cells, (C) cytotoxicity, (D) indoleamine 2,3-dioxygenase 1 (IDO1), (E) interferon-gamma, and (F) programmed cell death protein 1 (PD1).
