# Supplementary material for: Analysis of Genomic Alterations Associated with Recurrence in Early Stage HER2-Positive Breast Cancer
Source: Cancers (Basel). 2022 Jul 27;14(15):3650. doi: 10.3390/cancers14153650 (PMC9367395; doi:10.3390/cancers14153650)
Supplement: Supplementary file 1 [file cancers-14-03650-s001.zip › Supplementary Table S1.pdf]

Supplementary Table S1. Clinicopathological characteristics of eight controls without recurrence included in the gene expression analysis

**Variables**	**Controls without recurrence (n = 8)**<br>**No. (%)**
Age, Median (range)	48 (36–58)
Premenopause	4 (50)
BMI ≥25 kg/m^2	4 (50)
Presence of family history	1 (12.5)
Smoking	
Non or Ex-smoker	8 (100)
Presence of comorbidity	2 (25)
Tumor location	
Right	3 (37.5)
Left	5 (62.5)
Histology	
Invasive ductal	8 (100)
Hormone receptor status	
HR-negative	3 (37.5)
HR-positive	5 (62.5)
Pathological stage	
Stage II	5 (62.5)
Stage III	3 (37.5)
LN-positive	5 (62.5)
Histological grade	
G2	2 (25)
G3	6 (75)
Presence of LVI	3 (37.5)
Ki-67 labeling index	
≥20%	7 (87.5)
Type of Operation	
TM	4 (50)
Type of Adjuvant chemotherapy	
Anthracycline/ taxane	5 (62.5)
Non-anthracycline	3 (37.5)

BMI: body mass index; HR: hormone receptor; LN: lymph node; LVI: lymphovascular invasion; TM: total mastectomy.
