# Supplementary material for: Analysis of Genomic Alterations Associated with Recurrence in Early Stage HER2-Positive Breast Cancer
Source: Cancers (Basel). 2022 Jul 27;14(15):3650. doi: 10.3390/cancers14153650 (PMC9367395; doi:10.3390/cancers14153650)
Supplement: Supplementary file 1 [file cancers-14-03650-s001.zip › Supplementary Table S2.pdf]

Supplementary Table S2. Breast Cancer Signature scores between patients with recurrence and without recurrence.

Signatures	Nonrecurrence (Mean ± SD)	Recurrence (Mean ± SD)	P value
CD8 T cells	6.9 ± 0.9	5.9 ± 1.1	0.030
Cytotoxic cells	5.5 ± 0.9	4.5 ± 1.0	0.027
Cytotoxicity	5.3 ± 0.9	4.3 ± 1.0	0.017
IDO1	7.8 ± 0.9	6.5 ± 1.5	0.022
IFN-gamma	9.5 ± 0.6	8.8 ± 0.9	0.045
PD1	3.9 ± 1.0	3.0 ± 0.9	0.031
SOX2	2.3 ± 0.8	3.3 ± 1.5	0.030
