# Supplementary figures and images for: Analysis of Genomic Alterations Associated with Recurrence in Early Stage HER2-Positive Breast Cancer
Source: Cancers (Basel). 2022 Jul 27;14(15):3650. doi: 10.3390/cancers14153650 (PMC9367395; doi:10.3390/cancers14153650)

## Slide 1
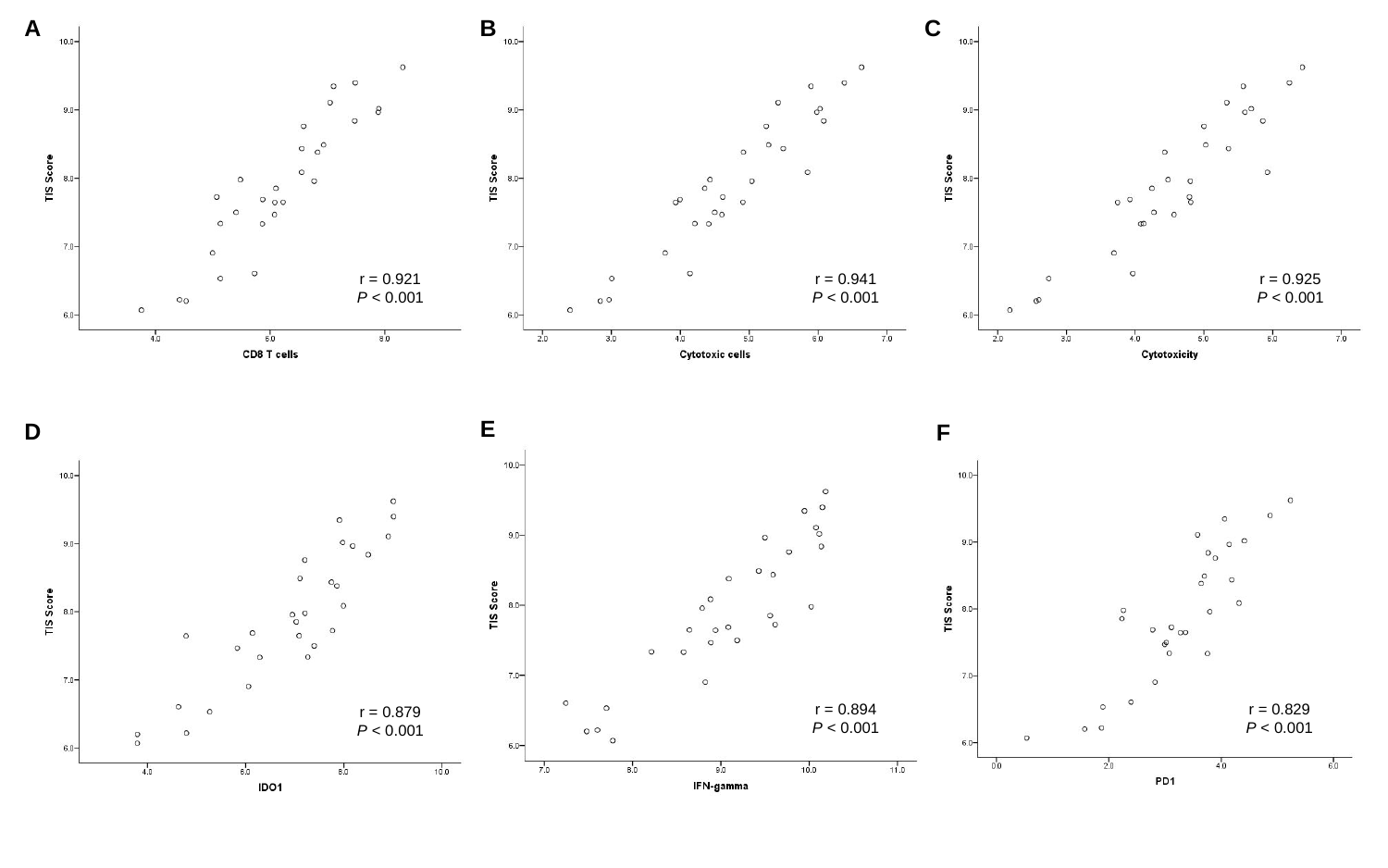

A
B
C
r = 0.921
P < 0.001
r = 0.941
P < 0.001
r = 0.925
P < 0.001
E
D
F
r = 0.894
P < 0.001
r = 0.829
P < 0.001
r = 0.879
P < 0.001

Supplement: Supplementary file 1 [file cancers-14-03650-s001.zip › Supplementary Figure S1.pptx]
